# Chaos and Robustness in a Single Family of Genetic Oscillatory Networks

**DOI:** 10.1371/journal.pone.0090666

**Published:** 2014-03-25

**Authors:** Daniel Fu, Patrick Tan, Alexey Kuznetsov, Yaroslav I. Molkov

**Affiliations:** 1 Department of Mathematics, Park Tudor School, Indianapolis, Indiana, United States of America; 2 Department of Mathematics, Carmel High School, Carmel, Indiana, United States of America; 3 Department of Mathematical Sciences, Indiana University-Purdue University of Indianapolis, Indianapolis, Indiana, United States of America; University of Pittsburgh, United States of America

## Abstract

Genetic oscillatory networks can be mathematically modeled with delay differential equations (DDEs). Interpreting genetic networks with DDEs gives a more intuitive understanding from a biological standpoint. However, it presents a problem mathematically, for DDEs are by construction infinitely-dimensional and thus cannot be analyzed using methods common for systems of ordinary differential equations (ODEs). In our study, we address this problem by developing a method for reducing infinitely-dimensional DDEs to two- and three-dimensional systems of ODEs. We find that the three-dimensional reductions provide qualitative improvements over the two-dimensional reductions. We find that the reducibility of a DDE corresponds to its robustness. For non-robust DDEs that exhibit high-dimensional dynamics, we calculate analytic dimension lines to predict the dependence of the DDEs’ correlation dimension on parameters. From these lines, we deduce that the correlation dimension of non-robust DDEs grows linearly with the delay. On the other hand, for robust DDEs, we find that the period of oscillation grows linearly with delay. We find that DDEs with exclusively negative feedback are robust, whereas DDEs with feedback that changes its sign are not robust. We find that non-saturable degradation damps oscillations and narrows the range of parameter values for which oscillations exist. Finally, we deduce that natural genetic oscillators with highly-regular periods likely have solely negative feedback.

## Introduction

Genetic oscillatory networks are networks of interacting proteins that regulate gene expression. They are found in many biological pathways, including the circadian rhythm [1], cell cycle regulation [2], apoptosis [3], metabolism [4], and morphogenesis [5], [6]. Such networks involve hundreds of reactions and thus are extremely difficult to characterize biologically and mathematically. This highlights the importance of methods to simplify the analysis of these networks.

One currently-utilized method for simplifying analysis is building a reduced mathematical model [7]–[10]. These models have significant value as they can be engineered biologically as artificial regulatory networks in the lab [11]–[15]. One type of reduced model, a delay differential equation (DDE), has demonstrated particularly strong potential as a viable method of analyzing genetic oscillatory networks [16]. DDEs account for time-consuming processes in the cell, such as slow nuclear transport and long chains of reactions, by incorporating a discrete time delay [17]. Consequently, DDEs are easier to interpret biologically than systems of ordinary differential equations (ODEs), which must account for each individual reaction with an additional differential equation.

From a mathematical standpoint, however, DDEs are significantly more complex than their ordinary counterparts. By construction, DDEs have an infinite number of dimensions. Consequently, they can exhibit high-dimensional dynamics. For example, while systems of ODEs require at least two equations to generate sustainable oscillations [18], a single DDE can produce both wildly complex behavior [19] and low-dimensional dynamics [17]. There is currently no analytical technique in the literature to predict the complexity of a DDE’s dynamics. In addition, it is not known what features determine whether DDEs exhibit robustness, the ability of a model to retain periodic oscillations against deterministic changes in the parameters of the equations. Because of these ambiguities, DDEs remain an area of active research [20], [21]. This highlights the need for further analysis of DDEs.

In our analysis, we examine models of the form:
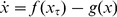
(1)where 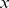 represents protein concentration, 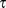 is a discrete time delay, 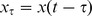, 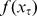 represents the synthesis of the protein, and 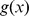 represents the degradation. This single-variable delay model accounts for the majority of minimal genetic oscillators modeled with delay [17], [22], [23]. Multi-variable delay models of minimal genetic regulatory oscillators have been reduced to single-variable delay models in previous studies [22]. Consequently, multi-variable delay models have been shown to exhibit properties that closely resemble those of single-variable delay models. Thus, our model covers a broad range of minimal genetic oscillators and gives us a comprehensive and accurate description of their dynamics.

In our study, we analyze the dynamics of DDEs of the form (1), determine which forms of the synthesis and degradation terms cause robustness, derive reduced systems of ODEs for robust models, and calculate analytic dimension lines for the non-robust models. In the Methods section, we outline the methods we use to achieve our aims. In the Results section, we present the results of our analyses. Finally, in the Discussion section, we discuss our findings and offer insights into their implications.

## Methods

### The Models

**Figure 1:**
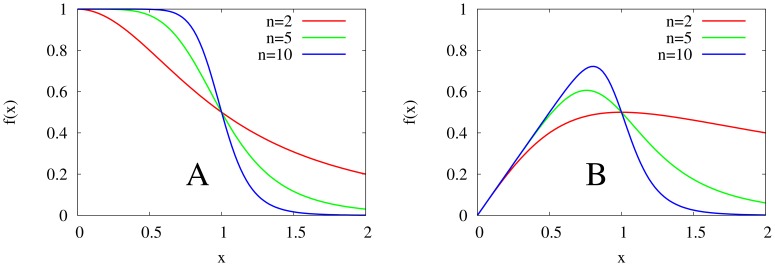
A graph of the synthesis terms 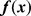 near 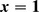 for varying 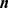. **A:** The monotonic synthesis term 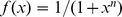. Because the term is monotonically decreasing, it represents universal negative feedback. Furthermore, as 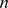 increases, 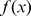 becomes increasingly step-like. **B:** The non-monotonic synthesis term 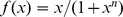. Because the term is not monotonically decreasing, it represents feedback that switches from positive to negative near 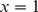. We have chosen to scale both graphs to 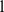 by setting 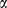 to 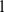.



For the dynamics of (1) to be applicable to genetic oscillators, a few conditions for 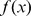 and 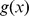 must be met. Both terms must be positive to ensure that they perform their intended biological roles. The degradation term must either be saturable [17] (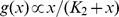) or non-saturable [19] (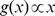). Furthermore, the synthesis term must either be monotonic, which corresponds to negative feedback (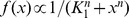), see Fig. 1A), or non-monotonic, which corresponds to positive feedback when 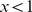 and negative feedback when 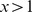 (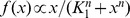, see Fig. 1B). For our analysis, we have elected to let the Michaelis constants, denoted by 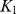 and 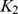, be equal (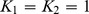) for ease of mathematical analysis. Furthermore, for the case where 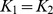, the degradation term is essentially non-saturable. For the case where 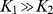, the degradation term is virtually constant, which means that the concentration of the protein is so high that proteosomes are always working at their maximal possible rate. This is not realistic biologically because a high copy number of the protein is typically hard to achieve technically and because proteosome saturation may impair other processes in the cell and cause cell death. Our preliminary analysis has also shown that the dynamics resulting from constant degradation are trivial: oscillations are not possible. Setting 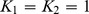 and pairing each of the two possibilities for 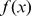 with the two possibilities for 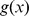 gives us the following family of four models:
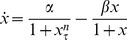
(2)


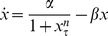
(3)




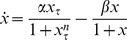
(4)




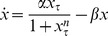
(5)where 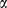 is the synthesis factor, 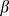 is the degradation factor, 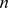 is the Hill cooperativity coefficient, 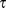 is a discrete time delay, and 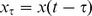. Of these four models, (2) [17], (4) [24], and (5) [19] have already been analyzed before, but for this study we wish to explore their properties further and in different contexts.

The analyses of these models involve examining properties related to their equilibrium states. We extend the definition of an equilibrium state for an ODE, which states that 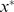 is an equilibrium state of the system 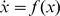 if and only if 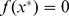, to DDEs. Our definition is as follows: 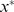 is an equilibrium state of the system 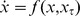 if and only if 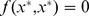. From this definition, we can derive the equilibrium states of the four models.

To start off, because the synthesis term 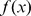 is monotonically decreasing and the degradation term 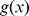 is monotonically increasing for both (2) and (3), we can see that each system has exactly one positive value 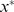 at which 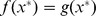. Therefore, we know that those two models each have exactly one positive equilibrium state. Next, we can see that (4) and (5) each have an equilibrium state 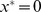. Additionally, (5) has an equilibrium state 
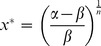
 for 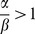, which is the system’s only other positive equilibrium state. Unfortunately, the other equilibrium states for (4) are much more dependant on the parameters 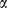 and 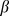, and we will not examine them in our analysis for that reason.

### Bifurcation Analysis

The first step in examining the properties of these genetic oscillators is to determine the values of the parameters at which oscillations appear. Such a change is a bifurcation, which is defined as a qualitative change in the dynamics of a system that results from a change in the parameters of the system. A bifurcation curve, which defines the values of the parameters at which bifurcations occur, can be calculated by performing a linear stability analysis [18] on (1).

To begin the derivation of the bifurcation curves, we linearize the system around the fixed point 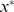 by letting 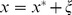, giving us the following linearized system via a Taylor series substitution:
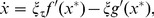
(6)where 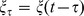. Next, we assume that the solution to (6) is of the form 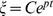 and substitute it into (6), giving us the following equation for 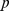:



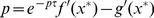
(7)We know that 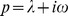, so, by substituting 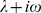 for 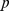 in (7), we can solve for 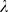 by converting from exponential form to CIS form and isolating the imaginary terms, which lets us arrive at (8). Next, we isolate the real terms of the CIS form of (7) after 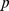-substitution and solve for 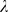 again, which gives us (9).
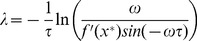
(8)

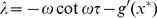
(9)


Now, we set 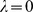 in (9) and solve for 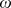 in (8), which we then substitute back into (9) to obtain (10), an equation for 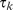 in terms of the number of pairs of positive 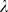
’s 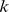 and the parameter 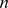. Finally, we solve for 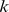 to obtain (11), which gives us the number of pairs of positive 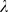
’s for a given 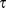 and 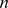.
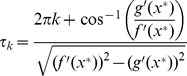
(10)




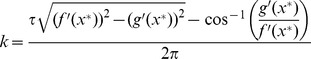
(11)


The curve given by substituting 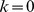 into (10) represents the bifurcation curve at which the first pair of characteristic exponents crosses the imaginary axis. This event marks a Hopf bifurcation, in which an equilibrium state loses stability and transforms into a stable limit cycle [18]. Because we have performed these calcuations on (1), we have derived general formulae that we can use to analyze (2)
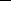
(5) by plugging in the specific forms of 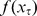 and 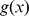 into (10) and (11).

### Numerical Details

In our numerical simulations, we generate time series using Euler’s method. We also tried using fourth-order Runge-Kutta (RK4), but it did not give any advantage for the purpose of calculating period, amplitude, or correlation dimension. We tested the stability of Euler’s method by choosing a few sets of parameters and choosing a time step for Euler’s method such that the maximal difference between RK4 and Euler’s method at each step was less than 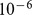. We found that a time step of 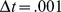 was sufficient.

To generate three-dimensional diagrams corresponding to how the period and amplitude of the oscillations respond to changes in both 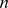 and 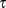, we generate a time series for some value of 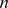 and 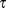. For this time series, we record a time 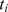 whenever 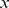 crosses 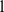 from above. We let the period of the oscillation for the 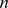 and 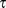 at 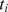 be the time difference between 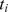 and 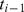, and we let the amplitude of the oscillation for the 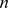 and 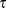 at 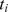 be the difference between the highest value of 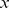 and the lowest value of 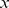 since 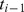. We then change 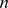 or 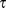 by a small value and then repeat the process until the full diagrams are generated.

Finally, although DDEs have infinite dimensionality, they often exhibit low-dimensional dynamics. To characterize the complexity of their dynamics, we need to numerically estimate the dimension of the system. The easiest way to numerically estimate the dimension from a one-dimensional time series is to numerically calculate the correlation dimension. To do this, we use the TISEAN package [25]. TISEAN calculates the correlation dimension 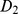 using the following formula:
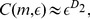
(12)where 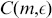 is the correlation sum, defined by the following formula:
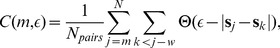
(13)where 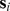 are 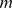-dimensional vectors, 
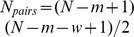
 is the number of pairs of points covered by the sum, 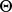 is the Heaviside step function [26], and 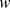 is the Theiler window [27]. To make the numerical estimation of the correlation dimension smoother, TISEAN furthermore calculates the Gaussian kernal correlation integral 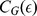, which can be obtained from 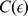 using the following formula:
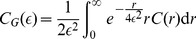
(14)



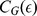
 has the same scaling properties as 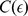, and it is from 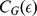 that the final correlation dimension is calculated. For more details and a deeper explanation on correlation dimension, see ref. [25].

### Reduction to Systems of ODEs

As discussed in the introduction, an area of particular interest is the synthesis of reduced models of the DDEs. Such reductions greatly reduce the complexity of the original models and allow for a substantially simpler analysis of their properties.

To reduce a system, we begin by converting the first-order DDE into a system of infinitely-many first-order ODEs by rewriting the coordinate 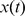 and its delayed counterparts 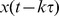 as a series of independent variables 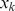 where 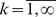. For models of the form (1), we get the recursive system 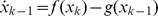.

The idea is to truncate this system at a certain 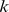. To do this, we first note that that the monotonic synthesis function becomes increasingly step-like, only taking on two values, as 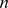 increases, see Fig. 1A. We take advantage of this fact to construct a switch variable 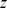 that will switch between those values. We then replace the synthesis term of the last equation by 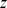, effectively eliminating all subsequent ODEs and creating a reduced system of ODEs. We must then consider the number of ODEs necessary to, in conjunction with a switching rule for 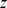, accurately reproduce the dynamics of the original delay system. Based on the number of ODEs we choose, we will have either a first-order or a second-order reduction.

#### First-Order Reduction

The minimum number of ODEs necessary to reproduce oscillations is one, since that corresponds to a two-dimensional system in 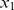 and 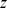. We call this a first-order reduction:
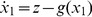
(15)


Instead of using the synthesis term 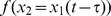 in (15), we replace it with 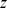. In the limit of 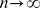, the monotonic synthesis function has two states, 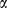 and 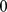. We therefore let 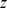 take on two states, 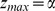 and 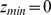. Suppose that at time 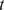, 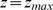. 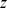 stays at this value as long as 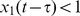. In this interval of low 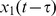, 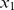 monotonically increases until 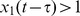. At that time, 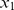 takes on the value 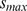 given by the following integral:
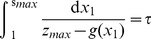
(16)


Thus, we switch 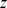 from 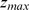 to 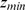 when 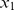 reaches the switching point 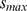. Similarly, 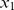 monotonically decreases when 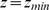 until 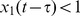. At that time, 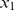 takes on the value 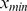 given by the following integral:
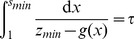
(17)


Again, we switch 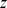 from 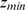 to 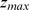 when 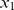 reaches the switching point 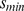. A consequence of switching 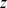 at 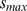 and 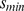 these switching points are upper and lower boundaries of the trajectory. This idea will become important when deriving the second-order reduction.

#### Second-Order Reduction

We hypothesize that we can achieve a more accurate approximation by increasing the number of ODEs to two. Consider a reduced system of two ODEs 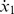 and 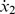. We call this the second-order reduction:
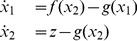
(18)


We let 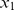 represent 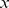 and 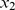 represent 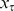 from the original DDE. Instead of replacing 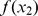 with 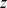 as in the first-order reduction, we replace 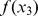 with 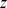. A major difference between the first and second-order reductions is in the treatment of 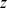. Since we have two dynamical variables 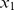 and 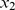, switching conditions for 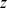 can depend on both of them. Accordingly, we will switch 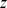 not at switching points as in the first reduction, but at switching curves which, similarly to the first-order reduction, can be derived as boundary curves for the trajectories of the DDE in a projection onto the 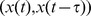 plane.

Let us denote the two values that the synthesis function switches between as 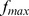 and 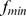. There are two boundary curves on the 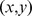 plane: a lower boundary that the curve must always stay to the right of, and an upper boundary that the curve must always stay to the left of. To calculate the lower boundary curve, we notice that 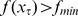 for all 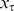. Since we are only dealing with positive protein concentrations, any solution 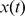 of (1) is greater than a solution of
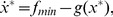
(19)assuming that 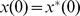. We can say that
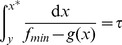
(20)defines the solution 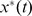 of (19) at time 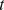 with the initial condition 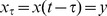. Accordingly, any solution of (1) such that 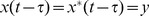 satisfies 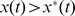. Thus, any trajectory of (1) lies to the right of the curve defined by (20) on the 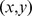 plane. To calculate the upper boundary curve, we notice that 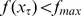 for all 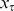. This means that any solution 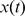 of (1) is less than a solution of
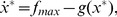
(21)assuming that 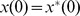. We can say that
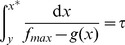
(22)defines the solution 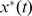 of (21) at time 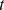 with the initial condition 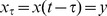. Using similar reasoning as above, any trajectory of (1) lies to the left of the curve defined by (22).

Because (20) and (22) define lower and upper boundary curves respectively, we need to switch 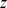 when the image point 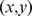 crosses either of the boundary curves. If 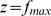 when 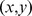 crosses a boundary curve, we will switch 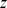 to 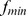; likewise, if 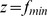 when 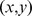 crosses a boundary curve, we will switch 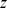 to 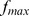.

Higher order reductions through adding additional dimensions may be possible. However, while there are qualitative improvements in the second-order reduction over the first-order reduction (which will be discussed in the results), we did not find a method for qualitatively improving the reduction in the space of higher dimensions. Since our study is primarily concerned with the qualitative characteristics of our models, we will not discuss higher-dimension reductions further in this study.

## Results

### Bifurcation Curves

Using the methods outlined in the section on Bifurcation Analysis, we calculate bifurcation curves for each of the four models. For the two models with monotonic synthesis terms, (2) and (3), 
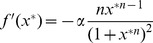
. For the two models with non-monotonic synthesis terms, (4) and (5), 
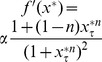
. For the two models with saturable degradation, (2) and (4), 
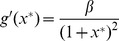
. For the two models with non-saturable degradation, (3) and (5), 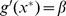. Substituting the values specified for 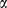 and 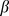 into these equations and substituting the resulting values into (10), we can calculate bifurcation curves for each of the models.

**Figure 2:**
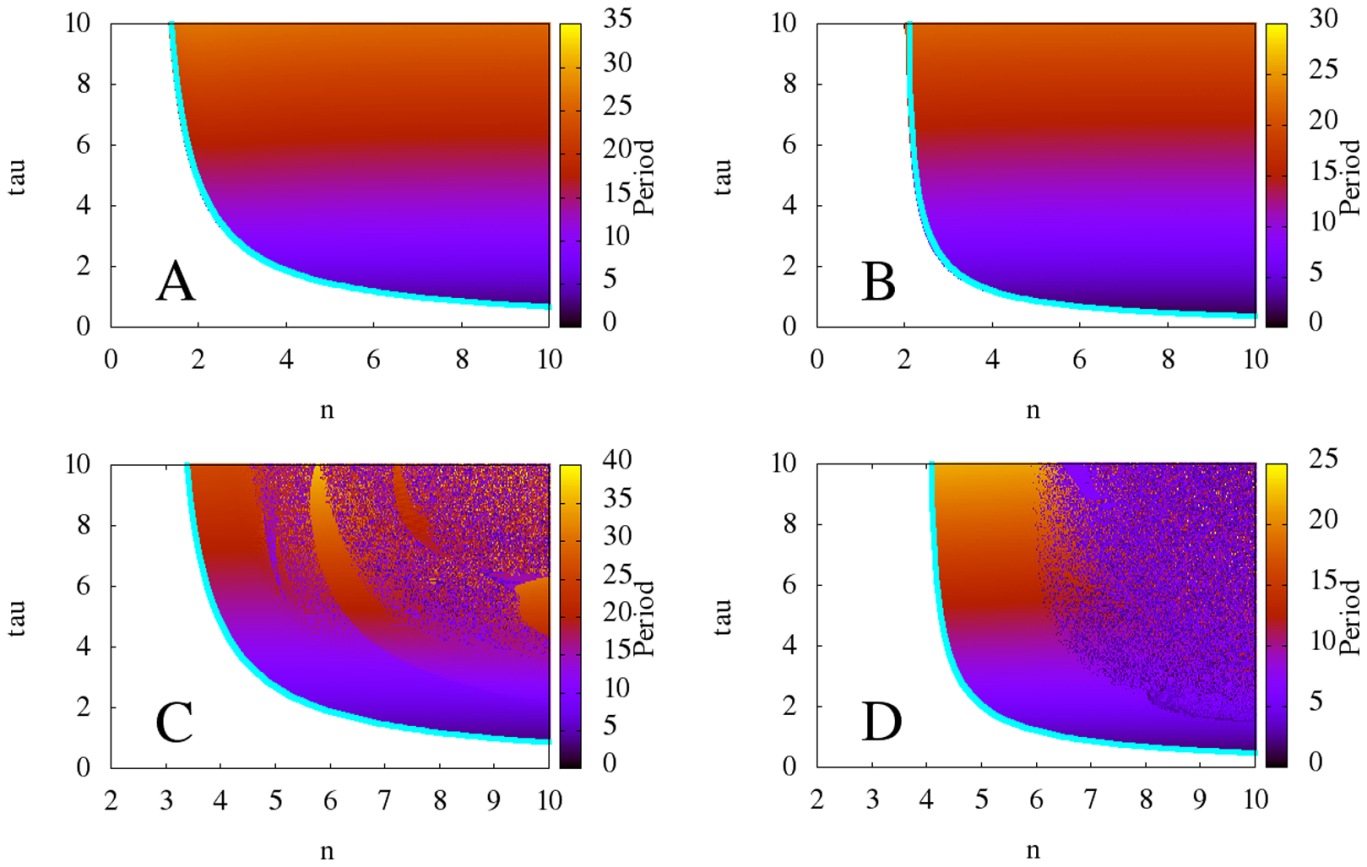
Two-dimensional diagrams showing the dependence of the period of oscillation of the models on 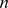 and 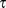, along with bifurcation curves in blue. The top two diagrams represent the models with monotonic synthesis, while the bottom two diagrams represent the models with non-monotonic synthesis. Similarly, the left two diagrams represent the models with saturable degradation, while the right two diagrams represent the models with non-saturable degradation. From these diagrams, it is apparent that the models with non-monotonic synthesis are not robust at high 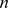 and 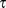, while the models with monotonic synthesis are robust at high 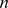 and 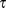. Note that the scales of the 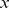-axes and color axes vary for each diagram. 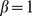 for all four models, 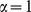 for the models with saturable degradation, and 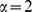 for the models with non-saturable degradation. 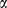 and 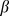 are chosen to keep the equilibrium state 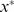 at 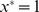.

As shown in Fig. 2, these bifurcation curves correspond to the the birth of oscillations, as predicted. Thus, (10) at 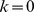 yields the equation of a bifurcation curve representing a Hopf bifurcation.

### Analysis of the Models

**Figure 3:**
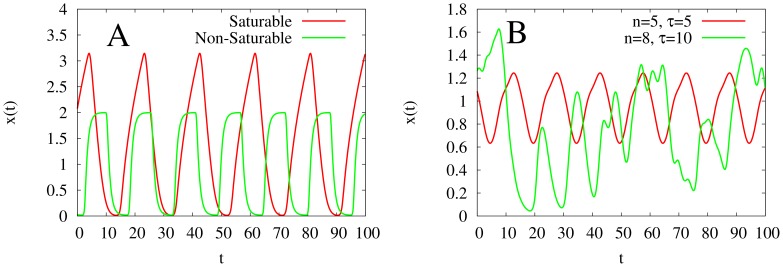
Various time series of the four models. **A:** Time series for the models with monotonic synthesis at 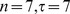. The model with saturable degradation is in red, and the model with non-saturable degradation is in green. **B:** Time series for the model with non-monotonic synthesis and saturable degradation at 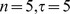 in red and at 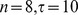 in green. Our simulations indicate that the models with monotonic synthesis stay robust at high 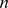 and 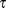, whereas the models with non-monotonic synthesis become chaotic at high 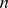 and 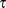. 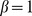 for all models, 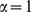 for the models with saturable degradation, and 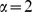 for the model with non-saturable degradation. 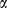 and 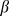 chosen to keep the equilibrium state 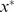 at 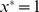.

In this section, we generate time series for the four models and discuss their behavior at different parameter values. In our simulations, we find that for each model, there are parameters at which the system produces regular, robust oscillations (see Fig. 3). However, increasing 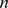 for the models with non-monotonic synthesis, (4) and (5), causes their dynamics to become drastically more complex and even chaotic.

**Figure 4:**
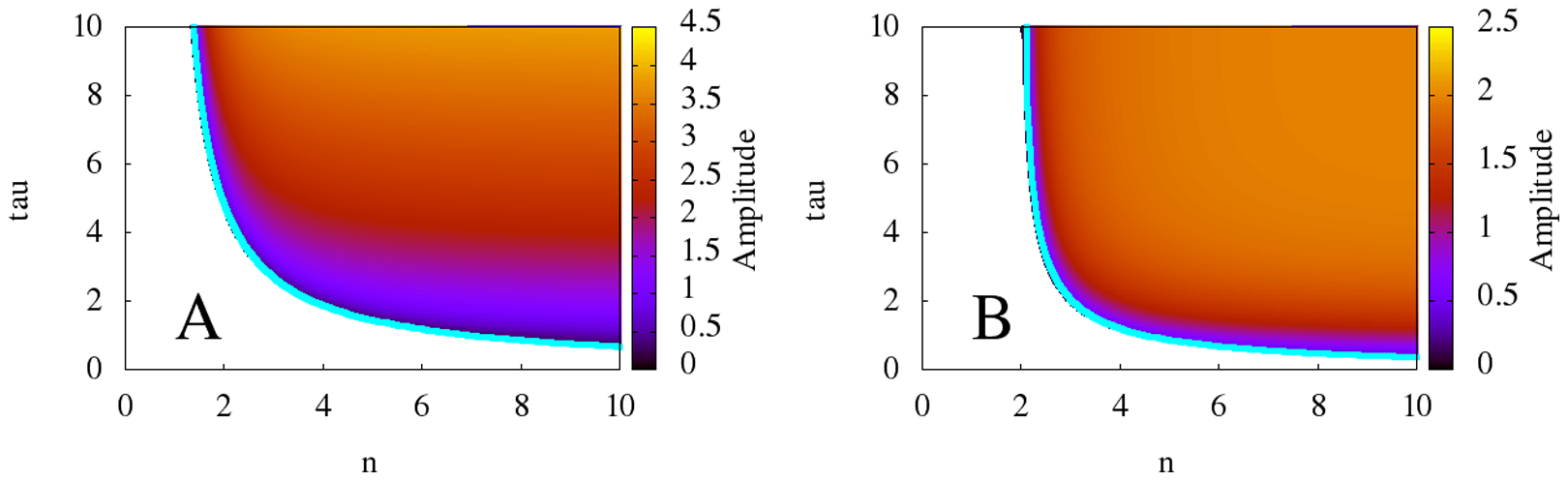
Two-dimensional diagrams showing the dependence of amplitude of oscillations of the models with monotonic synthesis with 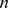 and 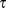, along with bifurcation curves in blue. **A:** the model with saturable degradation. **B:** the model with non-saturable degradation. Notice that the amplitude of the model with saturable degradation increases with 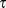, whereas the amplitude of the model with non-saturable degradation saturates. 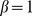 for both models, 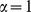 for the model with saturable degradation, and 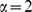 for the model with non-saturable degradation. 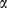 and 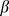 are chosen to keep the equilibrium state 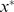 at 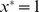.

To better understand the effects the parameters have on the dynamics of the models, we generate two-dimensional bifurcation diagrams for each of the models, observing how the period and the amplitude of the models’ oscillations change with 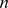 and 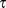. Our simulations indicate that the period of the oscillations increases linearly with 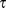 for all the models, as long as the parameter 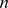 is such that the model does not exhibit high-dimensional chaotic behavior (see Fig. 3). However, the same is not true for the amplitude of the oscillations (see Fig. 4). The amplitude of the oscillations increases with 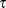 for (2). In contrast, the amplitude of the oscillations is largely constant for (3), despite increases in both 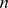 and 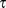.

**Figure 5:**
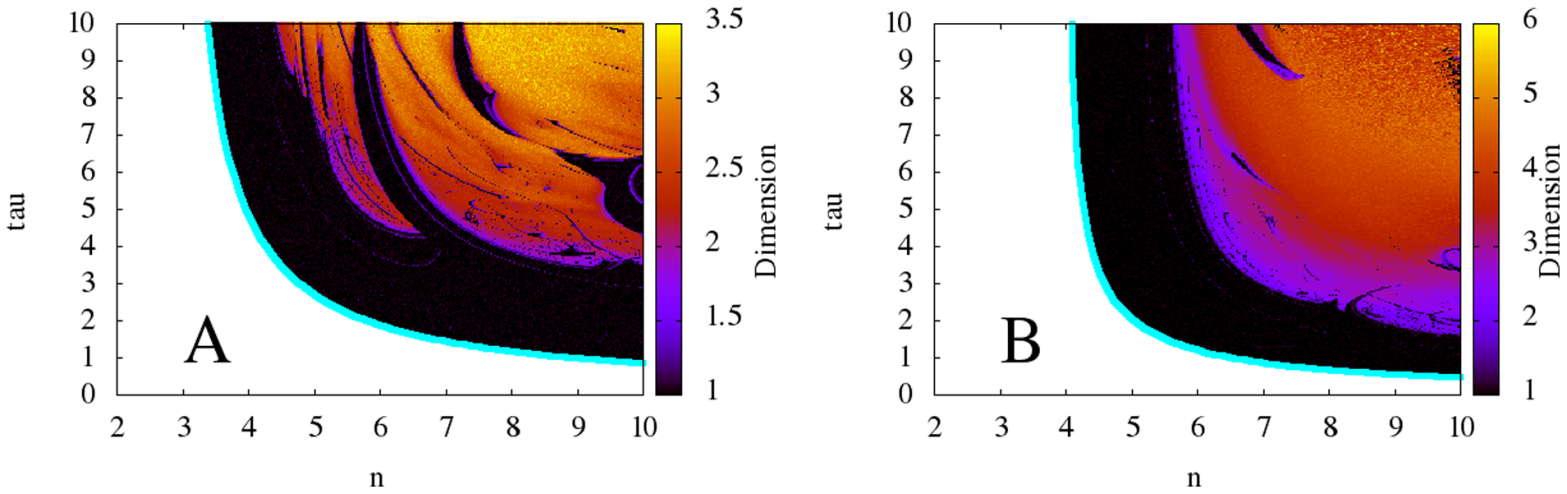
Two-dimensional diagrams showing the dependence of the correlation dimension of the time series obtained from the models with non-monotonic synthesis on 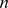 and 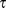, along with bifurcation curves in blue. **A:** the model with saturable degradation. **B:** the model with non-saturable degradation. The diagrams indicate that for high 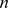 and 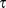, the models with non-monotonic synthesis exhibit high-dimensional, chaotic behavior. Note that the color axes vary between the two diagrams. 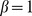 for all models, 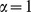 for the models with saturable degradation, and 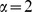 for the model with non-saturable degradation. 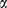 and 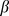 chosen to keep the equilibrium state 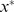 at 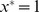.

Significantly, Fig. 5 shows that the models with non-monotonic synthesis exhibit high-dimensional, chaotic behavior for a large range of parameter values, whereas those models with monotonic-synthesis exhibit regular, periodic, robust oscillations for all values of 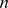 and 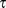 at which oscillations exist. This lets us conclude that the synthesis term determines whether the dynamics of genetic oscillatory models governed by DDEs of the form (1) become chaotic at high 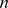 and 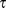.

Further analysis of the monotonic synthesis term 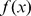 provides a clue regarding the reason the monotonic synthesis term yields robust, regular oscillations. Figure 1A shows the behavior of 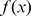 around 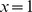. For values of 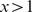, 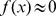, whereas for values of 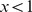, 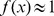. For large values of 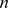, in fact, 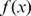 behaves very much like a stepwise function. This property of the monotonic synthesis term, coupled with the fact that the models with the monotonic synthesis term are robust make those models prime candidates for reduction via the methods outlined in the section on Reduction to Systems of ODEs.

### Reduction of Systems with Monotonic Synthesis

As discussed in the section on Reduction to Systems of ODEs, we can use the step-like nature of the monotonic synthesis term to reduce the models with monotonic synthesis to systems of ODEs.

#### Saturable degradation

We begin the first-order approximation of (2) by writing it in the form of (15):
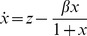
(23)


By substituting 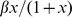 into (16) and (17), we can calculate the switching points, 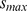 and 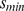 respectively, which satisfy the following equations:
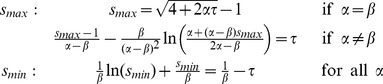
(24)


**Figure 6:**
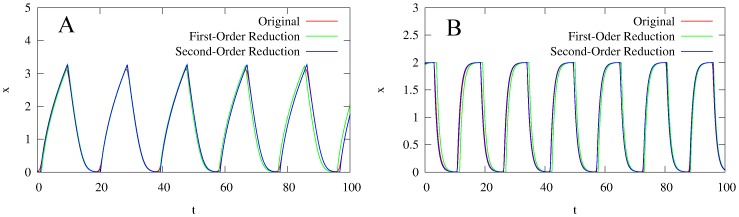
Time series of the two models with monotonic synthesis, along with their first- and second- order reductions, at 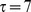 and two different values of 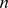 (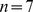 for A, 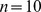 for B). **A:** the model with saturable degradation. **B:** the model with non-saturable degradation. In both figures, the red curve is the original model, the green curve is the first-order reduction, and the blue curve is the second-order reduction. For both models, both reductions approximate the originals well. However, the periods of the first-order reductions are slightly off from the originals, whereas the periods for the second-order reductions are much closer. 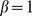 for all models, 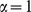 for the models with saturable degradation, and 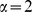 for the model with non-saturable degradation. 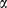 and 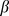 chosen to keep the equilibrium state 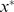 at 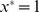.

**Figure 7:**
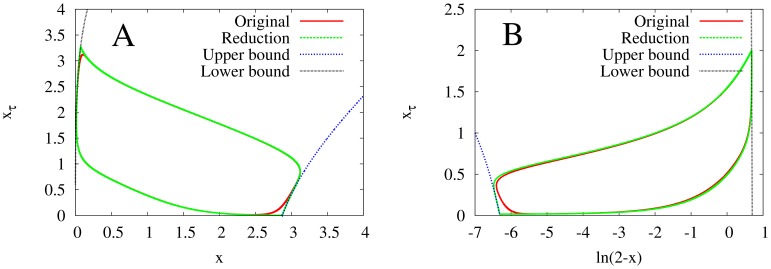
Dependence of the period on 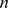, with 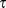 fixed at 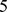. **A:** the model with saturable degradation. **B:** the model with non-saturable degradation. In both figures, the red curve is the period of the original model, the blue curve is the period of the first-order reduction, and the green curve is the period of the second-order reduction. For both pictures, the second-order reduction reproduces the dependence on the period on 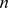 for sufficiently large 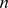. 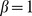 for all models, 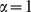 for the models with saturable degradation, and 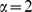 for the model with non-saturable degradation. 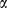 and 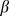 chosen to keep the equilibrium state 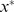 at 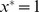.

Using these switching points, our simulations (see Fig. 6A) show that the first-order reduction approximates (2) well but does not retain a dependence of the period of oscillation on 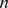 (see Fig. 7A).

For the second-order approximation, we begin by writing (2) as a system of ODEs 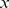 and 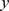, and switch variable 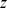:
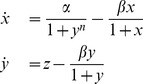
(25)where 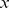 represents 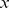 in the original DDE, 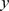 represents 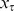 in the original DDE, and 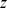 represents 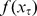. For this system, we let 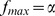 and 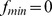, since it is not possible to calculate the maximum and minimum of the synthesis function any more precisely. We thus let 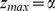 and 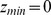. Substituting 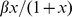 for 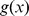 and 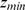 for 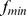 in (20), expanding, and substituting 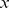 for 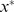 to map the curve to the 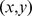 plane yields the following lower boundary curve:



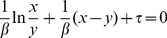
(26)Substituting into and expanding (22) yields the following upper boundary curve:
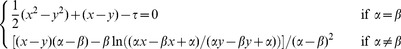
(27)


**Figure 8:**
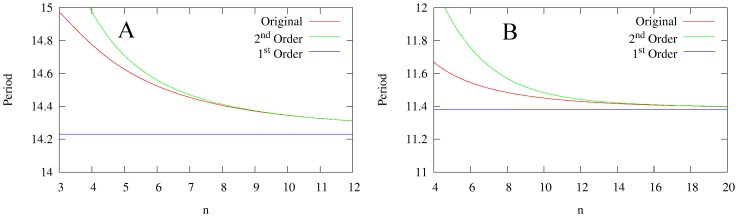
Phase portraits of the two models with monotonic synthesis, along with their second-order reductions to systems of ODEs, at 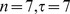. **A:** the model with saturable degradation. **B:** the model with non-saturable degradation. In both figures, the red curve is the original model, the green curve is the second-order reduction, and the blue dotted and black curves are switching curves. The closeness with which the second-order reductions approximate the originals shows that the second-order reduction technique is valid. Note that the 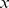-axes for the two graphs are different for better resolution. 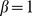 for all models, 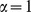 for the models with saturable degradation, and 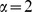 for the model with non-saturable degradation. 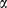 and 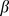 chosen to keep the equilibrium state 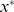 at 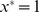.

Using these boundary curves, our simulations (see Figs. 6 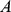 and 8A) indicate that (25) approximates (2) well for sufficiently high 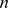. Furthermore, we find that the second-order reduction adds a correct dependence of the period on 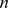 (*7* 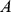).

#### Non-saturable degradation

To produce the first-order approximation for (3), we again begin by writing it in the form of (15):
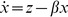
(28)


By substituting 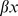 into (16) and (17), we can calculate the upper and lower boundaries, 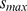 and 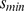 respectively, which satisfy the following equations:
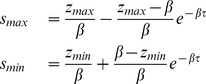
(29)


Using these switching points, our simulations (see Fig. 6B) show that the first-order reduction approximates (3) well. Once again, however, the first-order reduction provides no dependence of the period on 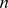 (see Fig. 7B).

For the second-order approximation, we again begin by writing (3) as a system of ODEs 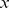 and 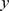, and switch variable 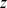:
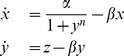
(30)where 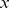 represents 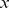 in the original DDE, 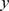 represents 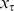 in the original DDE, and 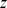 represents 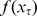 in the original DDE.

For the second-order reduction, we let 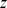 switch between two values that are close to 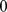 and 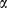 but are significantly different from them. Recall that 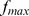 and 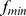 are the maximum and minimum values of the synthesis function 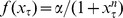. Because of the form of (30), 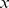, and therefore 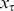 as well, are bounded from above by the maximum value of 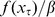 and from below by the minimum value of 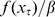. The maximum value of 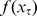 is in turn determined by the minimum value of 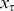, and the minimum value of 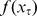 is determined by the maximum value of 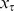. Therefore, 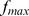 and 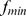 are the solutions of the following system:
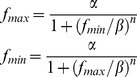
(31)


We numerically calculate 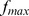 and 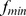 and let 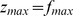 and 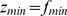. Substituting 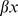 for 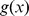 and 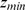 for 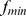 in (20), expanding, and substituting 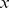 for 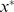 to map the curve to the 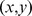 plane yields the following lower boundary curve:
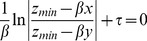
(32)


Substituting into and expanding (22) yields the following upper boundary curve:
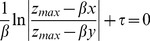
(33)


Using these boundary curves, our simulations indicate (see Figs. 6B and 8B) that the second-order reduction again approximates (3) well for sufficiently high 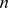. We also again find that the second-order reduction adds a correct dependence of the period on 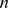 (see Fig. 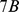).

For both models, increasing the order of the reduction from first to second-order introduces a qualitative improvement in the approximation. In neither of the first-order reductions is there a dependence of the period of oscillation on 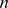. In fact, as Fig. 7 shows, both first-order reductions underestimate the period for all 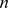. However, both second-order reductions provide an asymptotically correct dependence of period on 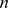 for large 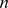 (about 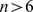 for (2) and 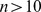 for (3)). This improvement confirms that the second-order reduction is an approximation of a higher precision than the first reduction.

### Dimension Analysis of Non-Robust Models

As discussed before, (4) and (5) display high-dimensional dynamics at increased values of 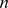 and 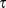. The dependence of the correlation dimension on parameters 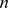 and 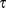 is shown in Fig. 5. We hypothesize that the dimension of the dynamics should be related to the number of conjugate pairs of characteristic numbers with positive real part. Our reasoning is largely geometrical. When the first pair of conjugate pairs of characteristic numbers crosses the imaginary axis, a limit cycle in one subsystem is born. When additional conjugate pairs cross the imaginary axis, limit cycles are born in additional dimensions. Since the motion of the trajectory is a result of the motion in all subsystems, additional conjugate pairs thus correspond to more complex behavior. In addition, because (11) gives the number of pairs 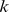 of characteristic numbers 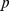 with positive real part, it should be related to the dimension.

We compute (11) for (4) and (5), which yields the following two equations, respectively:
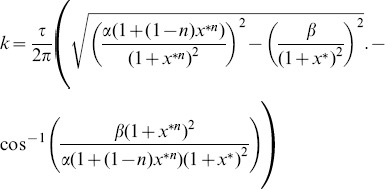
(34)




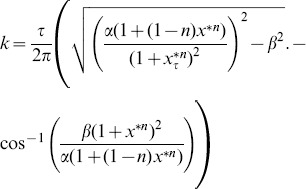
(35)


**Figure 9:**
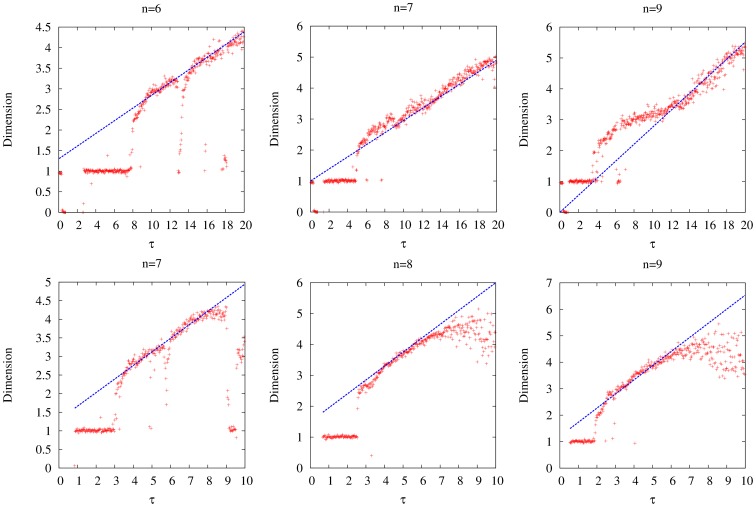
Diagrams showing the dependence of the numerically-calculated dimension of the trajectory of the models with non-monotonic synthesis on 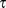, along with the analytical dimension lines, for different 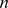. The set of diagrams on the top corresponds to the model with saturable degradation, and the set of diagrams on the bottom corresponds to the model with non-saturable degradation. The diagrams indicate that the slope of the analytical dimension lines match the slope of the numerically-calculated dimension points. It is important to note that the numerical estimates fail for high dimension, as evidenced by the trailing points in the bottom set of diagrams. The analytical dimension lines have no such limitation. 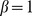 for all models, 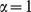 for the models with saturable degradation, and 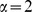 for the model with non-saturable degradation. 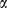 and 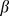 chosen to keep the equilibrium state 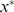 at 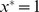.

If we take the above two equations and compare them to the calculated correlation dimensions of their respective models, we find that the slopes 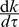 of (34) and (35) match the change in the correlation dimension with respect to 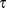. In Fig. 9, we take the lines given by the above two equations and manually adjust their offsets to show this.

## Discussion

We have developed two novel techniques for analyzing DDEs: a reduction of a DDE to a system of ODEs and an equation giving the rate of change of dimension. We have used these two techniques to analyze a family of four DDEs, each with a different combination of synthesis and degradation terms. In doing so, we have determined criteria for robustness as well as the roles of the synthesis and degradation terms within the family of four DDEs.

Our method for reducing models with step-like synthesis terms is, to the best of our knowledge, the first of its kind. The reduction allows us to analyze DDEs easier, for the reduced systems are only two- or three-dimensional, whereas the original DDEs are infinitely-dimensional. In particular, it allows us to make conclusions about the dynamics of the original models at high 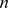 and 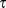, parameter ranges at which complex dynamics are expected to occur. Both reductions are robust at high 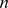 and 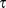, even though the second-order reduction has three variables and could therefore be chaotic. This leads us to believe that reducibility corresponds to robustness.

Our reduction method does have some limitations, however. In the first-order reduction, there is no accurate dependence of the period on 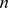, and the reduction tends to underestimate the period for low values of 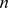. Our second-order reduction does introduce a dependence of the period on 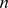, but it tends to overestimate the period for low values of 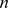. This suggests that potentially better higher order reductions may exist if more precise boundary curves can be derived in the space of a higher dimension.

Our analytical dimension estimates are, to the best of our knowledge, the first analytical method for estimating the rate at which the dimension of a system grows. This is significant because numerical estimates of dimension require exponentially longer time for accurate calculation as the dimension grows [25]. Additionally, numerical estimates have a tendency to fail at high dimensions (see Fig. 9). Our analytical estimates do not suffer from these numerical limitations. Our findings also predict that the dimension of the models with the non-monotonic synthesis term grows linearly with 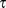.

A recent study has concluded that models with negative feedback are robust and hypothesized that models with feedback that switches its sign (called “mixed-mode feedback” in the study in question) might not be robust [17]. Our results support this. The models with the monotonic synthesis term, which corresponds to negative feedback, produce robust oscillations at lower levels of 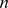 and sustain them for high levels of 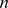 and 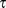. On the other hand, the models with the non-monotonic synthesis term, which corresponds to feedback that switches its sign near 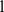, require higher levels of 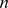 to produce oscillations and become chaotic at high 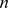 and 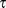. Furthermore, the models with the monotonic synthesis term are reducible, whereas the models with the non-monotonic synthesis term are not reducible. Thus, our findings strongly imply that models with exclusively negative feedback are robust, whereas models with mixed-mode feedback are not robust.

Our results also characterize the role that the degradation term plays in the models. In our simulations of models with the monotonic synthesis term, the amplitude in the model with saturable degradation increases with 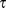, whereas the amplitude in the model with non-saturable degradation does not increase with 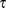. Although this phenomenon does not apply the models with the non-monotonic synthesis term, the average amplitude in the model with saturable degradation and non-monotonic synthesis is greater than the average amplitude in the model with non-saturable degradation and non-monotonic synthesis. Furthermore, through examining Fig. 2, it is clear that the bifurcation curves of the models with non-saturable degradation are steeper than those of the models with saturable degradation. Thus, we conclude that a non-saturable degradation term both damps oscillations and narrows the range of values of 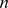 that can produce oscillations.

The bifurcation curves indicate that models of the form (1) require at least some degree of cooperativity to produce oscillations. Furthermore, some models become chaotic as the levels of cooperativity increase and cross further bifurcation curves 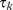 that correspond to 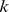, the number of conjugate pairs of characteristic exponents with positive real part, being greater than or equal to 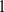. Thus, our results indicate that a certain degree of cooperativity is required for robust oscillations, but greater cooperativity can lead to chaos.

Our findings have implications for the role of feedback in natural genetic oscillators. Certain oscillators, such as the Circadian Clock, remain regular against a wide range of conditions [28], [29]. Since the monotonic synthesis term corresponds to negative feedback, and negative feedback results in robust oscillations at both low values of 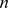 and high levels of 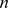 and 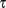, it is likely that natural genetic oscillators with highly-regular periods have monotonic promoters with negative feedback. Conversely, it is known that certain oscillators, such as heart rate or cell cycle, have slightly irregular period and near-constant amplitude. Previous research has shown these oscillators require both positive and negative feedback [30]. We have found that adding delay to oscillators with positive and negative feedback (i.e., having the non-monotonic synthesis term, see Fig. 1B) results in highly chaotic behavior. Thus, it is likely that any delay in heart rate and cell cycle oscillators is not large enough to play a significant role.

Finally, our findings have implications for the role of long chains of reactions, slow nuclear transport, etc, in natural genetic oscillators. Such processes take time and are thus equivalent to the delay in our models [17]. For oscillators with monotonic synthesis (and thus robust oscillations), such processes have exclusive control over the period of oscillation. Furthermore, for oscillators with monotonic synthesis and saturable degradation, the processes also have control over the amplitude of oscillations. On the other hand, for oscillators with non-monotonic synthesis, our analytic dimension lines indicate that the delays have direct control over the dimension of the model.

## Conclusions and Future Work

Our project has answered a number of questions concerning DDEs, but they have also highlighted a number of new research directions which could lead to further understanding of genetic oscillatory networks.

We have determined the effects the synthesis and degradation terms have on the end dynamics of the models, but understanding the fundamental mechanisms behind those effects could result in greater understanding of the models as a whole. For example, we have determined that the dimension of the systems with non-monotonic synthesis grows with 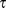 and that the dimension of the systems with monotonic synthesis does not. We do not yet have a compelling explanation for this, but further analysis of the synthesis and degradation terms might reveal the underlying reason. Next, we have empirically determined that the reductions of the robust models do not become chaotic, but we have not conducted a rigorous mathematical proof. Such a proof would likely involve taking a Poincáre section along one of the switching boundaries and could result in interesting new information about the reductions [18]. Similarly, the analytic dimension lines, which give the rate of change of dimension, are only the first step to having an analytical understanding of chaos in DDEs. Deriving a formula for the offsets of our analytical dimension lines would result in a complete analytical method for estimating dimension. In future projects, we would like to explore some of these research directions.
